# Effect of PRDX6 gene polymorphism on susceptibility to chronic obstructive pulmonary disease in the Chinese Han population

**DOI:** 10.1111/crj.13648

**Published:** 2023-06-17

**Authors:** Mingmei Xiong, Meihua Guo, Dongjian Huang, Jing Li, Yan Zhou

**Affiliations:** ^1^ Department of Critical Care Medicine The Third Affiliated Hospital of Guangzhou Medical University Guangzhou Guangdong China; ^2^ Guangdong Provincial Key Laboratory of Major Obstetric Diseases Guangzhou China; ^3^ Guangdong Provincial Clinical Research Center for Obstetrics and Gynecology Guangzhou China; ^4^ Department of Respiratory Guangzhou Chest Hospital Guangzhou China

**Keywords:** Chinese Han population, chronic obstructive pulmonary disease, PRDX6, single nucleotide polymorphisms, smoking

## Abstract

**Background:**

To explore the relationship of peroxiredoxin6 (PRDX6) tag‐single nucleotide polymorphisms (SNPs) with susceptibility to chronic obstructive pulmonary disease (COPD) in the Chinese Han population.

**Methods:**

A total of 502 patients with COPD and 481 healthy controls from nine hospitals in China were enrolled in this study. The PRDX6 tag‐SNPs were identified by linkage disequilibrium (LD) analysis in 30 healthy controls. The associations between identified tag‐SNPs and COPD risk were further evaluated.

**Results:**

Four PRDX6 tag‐SNPs, including rs7314, rs34619706, rs33951697, and rs4382766, were identified in 30 healthy controls. Moreover, in the allele model, there was no statistical difference in locus in PRDX6 between patients with COPD and healthy controls (*P* > 0.05). However, in the recessive model, rs33951697 locus in PRDX6 gene carrier with T/T had an increased risk of COPD (odds ratio [OR] = 2.59, 95% CI = 1.06–6.33, *P* = 0.028). Furthermore, in the relevance analysis between genetic polymorphisms and smoking behavior and lung function indexes, we found that the number of smoked cigarettes per day and FEV1/FVC differed among different genotypes of PRDX6, rs4382766, and rs7314 (*P* < 0.05).

**Conclusion:**

PRDX6 gene polymorphism with smoking status may contribute to the etiology of COPD in the Chinese Han population.

## Graphical abstract

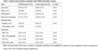

## 1 INTRODUCTION

Chronic obstructive pulmonary disease (COPD) is one of the major and increasingly prevalent health problems worldwide and the third leading cause of death in 2020.^1^ COPD is characterized by airflow limitation, which is associated with the abnormal inflammatory response of the lung to noxious particles or gases.^2^ COPD originates from airway epithelial cells that are the primary source of cigarette smoke inhalation.^3^ However, only about 20% of smokers have COPD, and their relatives are a high‐risk population, indicating that genetic susceptibility also contributes to the development of cancer.^4^, ^5^

Until recently, only mutations of the SERPINA1 gene that are responsible for a1‐antitrypsin deficiency are unambiguously associated with the development of COPD. However, this disorder accounts for only 1–2% of patients with COPD, and other disease‐associated alleles must exist. There are several identified risk loci.^6^, ^7^ Recently, they have identified 4 susceptibility loci that are associated with COPD, including 4q22 (FAM13A), 4q31 (HHIP), 15q25 (CHRNA3/CHRNA5/IREB2), and 19q13 (RAB4B, PRDX6, MIA, CYP2A6).^8^, ^9^, ^10^ Prdx6 was shown to be a powerful antioxidant enzyme, which was playing an important role in the pathogenesis of different chronic noncommunicable diseases including COPD.^11^

Therefore, we aimed to explore the relationship of PRDX6 with susceptibility to COPD. Moreover, by investigating COPD‐related clinical parameters, including lung function, smoking behavior, and emphysema scores, we differentiated more conclusively whether these gene variations directly affected the susceptibility to COPD or were actually mediated by risk factors of COPD such as smoking behavior, or even were just related to COPD subtypes.

## 2 SUBJECTS AND METHODS

### 2.1 Subjects

A total of 502 patients with COPD and 481 healthy controls from nine hospitals in China were enrolled in this study with informed consent. All participants were genetically unrelated ethnic Han Chinese. This study was approved by The Ethics Committee of Third Affiliated Hospital of Guangzhou Medical University and all the other local Ethics committees.

COPD was defined as the ratio of forced expiratory volume in 1 s (FEV1) to forced vital capacity (FVC) less than 0.7, and the predicted value of FEV1 after inhalation of β‐agonist was less than 80%. Patients with other significant respiratory diseases were excluded according to the chest X‐ray test. The healthy controls were volunteers who came to the hospital for physical examination. The inclusion criteria for controls were as follows: (1) people with normal lung function; (2) patients without known medical illnesses; (3) patients without pulmonary disease or other chronic diseases including lung cancer, pulmonary fibrosis, bronchiectasis, and so on; (4) FEV1/FVC < 70% (after inhaling bronchodilator). Subjects with chronic lung diseases or a family history of COPD in the control group were excluded. People who smoked <100 lifetime cigarettes are called nonsmokers; instead, smokers.

### 2.2 Detection of general clinical characteristics

Body mass index (BMI), prior employment, smoking history, and personal and family health histories were collected. As part of the referral process, pulmonary function tests, chest X‐ray, chest high‐resolution computed tomography (HRCT), and blood samples were obtained. Clinical analyses were carried out based on the Global Initiative for GOLD criteria.^12^

### 2.3 SNP selection and genotyping

Single nucleotide polymorphisms (SNPs) were screened from the noncoding region of PRDX6 if minor allele frequency > 5% from dbSNP and 4 SNPs (rs7314, rs34619706, rs33951697, and rs4382766) were finally selected. Blood samples were acquired from all subjects, and genomic DNA was extracted from peripheral blood leukocytes using a Blood & Cell Culture DNA Kit (QIAGEN). After measuring DNA concentration by NanoDrop 2000 nucleic acid quantitative instrument (Thermo Scientific, USA), genotyping was carried out by Genesky (Shanghai, China) using the SNPseqTM method. Quality control measures included that the genotype detection rate of all four SNPs was 99%. Approximately 5% of the samples were genotyped in duplicate to check for consistency. Moreover, there was no SNP deviated from Hardy–Weinberg equilibrium. The management and analysis of all genotyping data were performed using the BLAST‐Like Alignment Tool software.

### 2.4 Statistical analysis

Data in COPD and healthy controls were compared using the *χ* ^2^ test and Student's *t* test. The Hardy–Weinberg equilibrium for SNPs was also evaluated by the *χ* ^2^ test. Quantitative statistical analysis of age, packyear of smoking, and pulmonary function was calculated with Student's *t* test. Associations between the alleles of selected SNPs and the risks of COPD were assessed using the SHEsis software platform (http://analysis.bio-x.cn/myAnalysis.php).^13^ Differences in the distribution of genotypes under five genetic models, including dominant, recessive, overdominant, codominant, and log‐additive models were estimated by using the website software program SNPStats, and odds ratios (ORs) and 95% CIs were calculated by unconditional logistic regression analyses after adjusting for age, sex, and packyear of smoking.^14^ Akaike's information criterion was used to determine the best‐fitting model for each SNP.^15^ Stratification analysis was also carried out by variables of interest. The pairwise linkage disequilibrium (LD) among the SNPs was calculated using standardized coefficient *D*′, and haplotype blocks were defined as previously described.^16^ For haplotype analysis, we chose the SNPs in the same haplotype blocks analyzed by Haploview to estimate the associations between haplotypes and the risks of COPD using SNPStats. Analysis of variance (ANOVA) was used to analyze the clinical parameters such as emphysema scores. *P* < 0.05 was considered a significant difference, and all statistical analyses were performed using Stata version 13.0.

## 3 RESULTS

### 3.1 Clinical characteristics

A total of 502 COPD patients and 481 healthy people were recruited. The demographic and baseline clinical characteristics were presented in Table 1. The age of patients with COPD was older than healthy controls (*P* < 0.01). Patients with COPD had longer packyears of smoking than healthy controls (*P* < 0.01). Gender, smoking status, and BMI were similar (*P* > 0.05). Thus, age might be a confounding factor that may lead to bias, and we need to recognize and exclude the interference to statistical results in analysis. Patients with COPD had worse lung function than healthy controls, including lower FEV1 percent predicted (37% vs. 96%), FEV1 (0.93 vs. 2.51), FVC (2.25 vs. 3.10), and FEV1/FVC (0.41 vs. 0.81).

**TABLE 1:** Clinical characteristics of patients with COPD and healthy controls.

Items	COPD group (*n* = 502)	Control group (*n* = 481)	*P* values
Age (year)	68.76 ± 7.94	56.65 ± 9.52	<0.01
Male, *n* (%)	424 (84)	395 (82)	>0.05
BMI (kg/m^2^)	23.78 ± 2.39	24.00 ± 2.44	>0.05
Packyear of smoking	42.74 ± 27.23	35.69 ± 22.10	<0.01
Smoking status			
Smoker, *n* (%)	376 (75)	390 (77)	>0.05
Non‐smoker, *n* (%)	126 (25)	114 (23)	
Spirometry			
FEV1 (L)	0.93 ± 0.43	2.51 ± 0.50	<0.01
FEV1 (% predicted)	0.37 ± 0.17	0.96 ± 0.14	<0.01
FVC (L)	2.25 ± 0.69	3.10 ± 0.66	<0.01
FEV1/FVC, ratio	0.41 ± 0.12	0.81 ± 0.09	<0.01

*Note*: Data are presented as means ± SD. *P* < 0.05 indicates statistical significance.

Abbreviations: BMI, body mass index; packyears = (number of cigarettes smoked per day multiplied by number of years smoked)/20; COPD, chronic obstructive pulmonary disease; FEV1, forced expiratory volume in 1 s; FVC, forced vital capacity.

### 3.2 Genotype and allele frequencies of 4 SNPs of PRDX6

A total of 4 SNPs of PRDX6, including rs7314, rs34619706, rs33951697, and rs4382766, were genotyped in 502 patients with COPD and 481 healthy controls using the SNPseqTM method. The details of each SNP were presented in Table 2. All tested SNPs deviated from the expectation of Hardy–Weinberg equilibrium, and it showed no significant differences between patients with COPD and healthy controls (*P* > 0.05). As shown in Table 2, allele or genotype frequencies differed between COPD patients and healthies at rs33951697 (allele: *P* = 0.099, OR = 1.22, and 95% CI = 0.96–1.54; genotype: *P* = 0.01).

**TABLE 2:** Distributions of the PRDX6 SNPs in patients with COPD and healthy controls.

SNP	Genes	Groups	Gene type (fre%)			*P*	HWE	Allele		*P*	OR [95%]
rs7314	prdx6		T/T	C/T	C/C			C	T		
		COPD	72 (0.15)	232 (0.5)	164 (0.35)	0.591	0.5	560 (0.6)	376 (0.4)	0.364	0.92
		Control	75 (0.16)	237 (0.52)	146 (0.32)		0.2	529 (0.58)	387 (0.42)		[0.76–1.10]
rs33951697	prdx6		C/C	C/T	T/T			C	T		
		COPD	229 (0.51)	179 (0.4)	37 (0.08)	0.01*	0.81	637 (0.72)	253 (0.28)	0.099	1.22
		Control	165 (0.54)	133 (0.43)	9 (0.03)		0.0032	463 (0.75)	151 (0.25)		[0.96–1.54]
rs34619706	prdx6		A/A	A/G	G/G			A	G		
		COPD	248 (0.49)	217 (0.42)	46 (0.09)	0.968	0.88	713 (0.7)	309 (0.3)	0.799	0.98
		Control	241 (0.48)	216 (0.43)	47 (0.09)		0.89	698 (0.69)	310 (0.31)		[0.81–1.18]
rs4382766	prdx6		C/C	C/T	T/T			C	T	0.165	
		COPD	175 (0.34)	257 (0.5)	79 (0.15)	0.363	0.33	607 (0.59)	415 (0.41)		0.88
		Control	154 (0.31)	260 (0.52)	90 (0.18)		0.27	568 (0.56)	440 (0.44)		[0.74–1.05]

Abbreviations: CI, confidence interval; COPD, chronic obstructive pulmonary disease; HWE, Hardy–Weinberg equilibrium; ID, G base insertion/deletion polymorphism; OR, odds ratio; SNPs, single nucleotide polymorphisms.

* *P* < 0.05 represents significant difference.

### 3.3 Association of genotypes with COPD

Genotype frequencies of rs33951697 were compared with five genetic models (Table 3), respectively. A significant difference was observed in the best‐fitting Recessive model (*P* = 0.028). However, no significant associations between rs148832191 and rs11547373 were observed under any of the five genetic models (*P* > 0.05).

**TABLE 3:** Association between PRDX6 SNPs and the risk of COPD under different genetic models.

SNP	Model	Genotype	Control	COPD	OR (95% CI)	*P*
rs33951697	Codominant	C/C	165 (53.8%)	229 (51.5%)	1	0.09
PRDX6		C/T	133 (43.3%)	179 (40.2%)	1.01 (0.70–1.46)	
		T/T	9 (2.9%)	37 (8.3%)	2.60 (1.05–6.47)	
	Dominant	C/C	165 (53.8%)	229 (51.5%)	1	0.55
		C/T‐T/T	142 (46.2%)	216 (48.5%)	1.11 (0.78–1.59)	
	Recessive	C/C‐C/T	298 (97.1%)	408 (91.7%)	1	0.028*
		T/T	9 (2.9%)	37 (8.3%)	2.59 (1.06–6.33)	
	Over dominant	C/C‐T/T	174 (56.7%)	266 (59.8%)	1	0.72
		C/T	133 (43.3%)	179 (40.2%)	0.94 (0.65–1.34)	
	Log‐additive	‐	‐	‐	1.02 (0.90–1.64)	0.19
rs4382766	Codominant	C/C	154 (30.6%)	175 (34.2%)	1	0.22
PRDX6		C/T	260 (51.6%)	257 (50.3%)	0.75 (0.53–1.05)	
		T/T	90 (17.9%)	79 (15.5%)	0.91 (0.58–1.45)	
	Dominant	C/C	154 (30.6%)	175 (34.2%)	1	0.14
		C/T‐T/T	350 (69.4%)	336 (65.8%)	0.78 (0.57–1.08	
	Recessive	C/C‐C/T	414 (82.1%)	432 (84.5%)	1	0.6
		T/T	90 (17.9%)	79 (15.5%)	1.09 (0.72–1.64)	
	Over dominant	C/C‐T/T	244 (48.4%)	254 (49.7%)	1	0.088
		C/T	260 (51.6%)	257 (50.3%)	0.77 (0.57–1.04)	
	Log‐additive	‐	‐	‐	0.91 (0.73–1.14)	0.42
rs7314	Codominant	C/C	146 (3.9%)	164 (35%)	1	0.53
PRDX6		T/C	237 (51.8%)	232 (49.6%)	0.84 (0.59–1.19)	
		T/T	75 (16.4%)	72 (15.4%)	1.01 (0.63–1.64)	
	Dominant	C/C	146 (31.9%)	164 (35%)	1	0.44
		T/C‐T/T	312 (68.1%)	304 (65%)	0.88 (0.63–1.22)	
	Recessive	C/C‐T/C	383 (83.6%)	396 (84.6%)	1	0.59
		T/T	75 (16.4%)	72 (15.4%)	1.13 (0.73–1.74)	
	Over dominant	C/C‐T/T	221 (48.2%)	236 (50.4%)	1	0.26
		T/C	237 (51.8%)	232 (49.6%)	0.84 (0.61–1.14)	
	Log‐additive	‐	‐	‐	0.97 (0.77–1.22)	0.8
rs34619706	Codominant	A/A	241 (47.8%)	248 (48.5%)	1	0.38
PRDX6		A/G	216 (42.9%)	217 (42.5%)	0.85 (0.62–1.16)	
		G/G	47 (9.3%)	46 (9%)	1.18 (0.68–2.04)	
	Dominant	A/A	241 (47.8%)	248 (48.5%)	1	0.47
		A/G‐G/G	263 (52.2%)	263 (51.5%)	0.90 (0.66–1.21)	
	Recessive	A/A‐A/G	457 (90.7%)	465 (91%)	1	0.36
		G/G	47 (9.3%)	46 (9%)	1.28 (0.75–2.17)	
	Over dominant	A/A‐G/G	288 (57.1%)	294 (57.5%)	1	0.21
		A/G	216 (42.9%)	217 (42.5%)	0.82 (0.61–1.12)	
	Log‐additive	‐	‐	‐	0.98 (0.78–1.24)	0.88

*Note*: *P* values were calculated by unconditional logistic regression adjusted for age, sex, and packyear of smoking.

Abbreviations: COPD, chronic obstructive pulmonary disease; OR, odds ratio; SNPs, single nucleotide polymorphisms.

* *P* < 0.05 represents significant difference.

### 3.4 Relationship between gene polymorphism and lung function

Table 4 listed the SNP sites with significant differences in COPD lung function under different genotypes. It showed that the different genotypes of FEV1/FVC at rs4382766 and rs7314 of the PRDX6 gene have statistical differences (*P* < 0.05).

**TABLE 4:** Association of allele frequencies with lung function.

SNP	Gene types			*P*
PRDX6 rs4382766	C/C	C/T	T/T	
FEV1	0.92	0.88	0.86	0.581
FEV1 (%predicted)	0.37	0.35	0.34	0.707
FVC	2.18	2.28	2.23	0.43
FEV1/FVC	0.42	0.39	0.39	0.021*
PRDX6 rs7314	T/C	C/C	T/T	*P*
FEV1	0.88	0.93	0.87	0.579
FEV1,%predicted	0.35	0.37	0.35	0.467
FVC	2.29	2.20	2.19	0.476
FEV1/FVC	0.39	0.42	0.39	0.047*

Abbreviations: FEV1, forced expiratory volume in 1 s; FVC, forced vital capacity; SNP, single nucleotide polymorphism.

* *P* < 0.05 represents significant difference.

### 3.5 Association of allele frequencies with the severity of COPD

COPD patients were divided into two groups based on the pulmonary function tests (FEV1% predicted ≥red as GOLD I and II group and FEV1% predicted <50% as GOLD III and IV group). The genotype and allele frequencies of the 4 SNPs in both two groups were presented in Table 5. Allele and genotype frequencies differed between the two COPD groups at all 4 SNPs (*P* > 0.05).

**TABLE 5:** Distributions of the PRDX6 SNPs in COPD patients and association of allele frequencies with the severity of COPD.

SNP	Gene types	COPD		*P*	Dominant	*P*	Recessive
		GOLD I–II	GOLD III–IV		0R (95% CI)		0R (95% CI)
rs33951697	C/C	36	125	0.87	1.05 [0.62–1.77]	0.606	0.79 [0.32–1.95]
	C/T	28	107				
	T/T	7	20				
rs7314	T/C	33	136	0.378	1.28 [0.74–2.20]	0.881	1.06 [0.51–2.17]
	C/C	27	86				
	T/T	11	43				
rs4382766	C/T	36	150	0.326	1.30 [0.77–2.22]	0.719	1.14 [0.56–2.32]
	C/C	28	90				
	T/T	11	47				
rs34619706	A/G	37	119	0.155	0.69 [0.41–1.15]	0.591	0.77 [0.29–2.01]
	A/A	32	149				
	G/G	6	19				

Abbreviations: CI, confidence interval; COPD, chronic obstructive pulmonary disease; OR, odds ratio; SNPs, single nucleotide polymorphisms.

### 3.6 Association between PRDX6 polymorphism and COPD by smoking status‐stratified analysis

As shown in Table 6, the number of smoking per day (CPD) of different genotypes of PRDX6 at rs4382766 and RS7314 had statistical differences (*P* < 0.05).

**TABLE 6:** Association between PRDX6 polymorphism and COPD by smoking status‐stratified analysis.

SNP	Gene types			*P*
PRDX6 (rs4382766)	C/C	C/T	T/T	
CPD	19.20	22.91	17.51	0.013*
Packyear	45.128261	41.79	46.82	0.3737
PRDX6 (rs7314)	T/C	C/C	T/T	
CPD	23.34	18.90	17.71	0.006*
Packyear	41.17	44.52	45.37	0.456

Abbreviations: COPD, chronic obstructive pulmonary disease; CPD, the number of smoking per day; packyear, (number of cigarettes smoked per day multiplied by number of years smoked)/20; SNP, single nucleotide polymorphism.

* *P* < 0.05 represents significant difference.

### 3.7 LD and haplotype association analysis

Haplotype is a group of interrelated SNPs located in a specific region of a chromosome, and it tends to be inherited as a whole by offspring. Because the haplotype contains the genetic information of multiple SNPs, the analysis of haplotypes composed of a combination of multiple variant sites is better than the analysis of a single SNP in the research of complex diseases. In this study, based on genotyping data of healthy controls, Haploview 4.2 software was used to calculate the degree of linkage imbalance between tag SNP loci, and the linkage between loci was usually shown by the LD diagram. Among them, three sites on the PRDX6, including rs34619706, RS4382766, and rs7314, constituted a haplotype domain. These loci within the same haplotype domain have a high level of LD. An unconditional logistic regression analysis model was carried out to measure the association between the locus of the same haplotype domain and the risk of COPD according to the genotyping data of all subjects. In this study, an unconditional logistic regression analysis was performed on the tag SNP locus on the PRDX6 gene in the Chinese Han population. After adjusting gender, age, and smoking package years, no positive haplotype associated with COPD was found (*P* > 0.05). The analysis results of this haplotype were shown in Table 7.

**TABLE 7:** Association between PRDX6 haplotype frequency and COPD risk.

Haplotype	rs34619706	rs4382766	rs7314	Freq	OR (95% CI)	*P*_adjust_
1	A	T	T	0.4032	1	‐
2	G	C	C	0.3001	1.01 (0.77–1.31)	0.96
3	A	C	C	0.271	1.11 (0.85–1.46)	0.44
4	A	T	C	0.018	0.41 (0.16–1.02)	0.054
rare	*	*	*	0.0077	0.79 (0.24–2.61)	0.71

*Note*: The asterisk in the table means blank.

Abbreviations: COPD, chronic obstructive pulmonary disease; OR, odds ratio.

## 4 DISCUSSION

In this study, we evaluated the association between PRDX6 tag‐SNPs and susceptibility to COPD in the Chinese Han population. We found that PRDX6 polymorphisms and their interactions with smoking status may induce the etiology of COPD. Genetic variants in PRDX6 may affect pulmonary function indexes.

COPD can lead to dyspnea. It is caused by long‐term lung injury caused by smoking. Chronic pulmonary and systemic oxidative stress exists in patients with COPD, which can promote inflammatory response, autoimmune response, glucocorticoid resistance, accelerate lung aging, and increased airway hyperresponsiveness.^17^, ^18^ Oxidative stress has been recognized as an important predisposing factor for COPD. PRDX6 is an important antioxidant enzyme in the body, with the activity of glutathione peroxidase and phospholipase A2. It can defend against oxidative stress and repair membrane damage. PRDX6 has a high content in the lungs, especially in alveolar macrophages, neutrophils, Clara cells, and type II alveolar epithelial cells. Studies have confirmed that the combination of NRF2 and the antioxidant response element in the PRDX6 promoter region promotes the transcriptional expression of PRDX6.^19^ Muc5ac is a major component of mucus and can be activated by ROS. PRDX6 highly expresses in airway epithelium and protects the airway from oxidative stress. PRDX6 can decrease LPS‐induced Muc5ac increase.^20^

To further explore the relationship between PRDX6 and COPD, Sundar et al. demonstrated through in vivo animal experiments that after a short period of exposure to cigarette smoke, PRDX6 knockout mice would not accumulate inflammatory cells in large numbers, and the levels of pro‐inflammatory factors in the lungs did not change compared with those in the wild mice.^21^ However, the levels of antioxidant enzymes in their lungs were increased in wild mice. However, the levels of antioxidant enzymes in mice overexpressing the PRDX6 gene were relatively low. The above results indicated that PRDX6 targeted knockout did not aggravate the pulmonary inflammation but can increase the body's antioxidant enzyme levels, suggesting that PRDX6 can play an antioxidant role in COPD. Therefore, we suspected that PRDX6 polymorphisms may play an important role in the pathogenesis of COPD.

Although some genetic risk variants of COPD have been gradually discovered, most studies have focused on European Caucasians. Due to the differences in the prevalence and gene variants of COPD in different ethnic groups, we explored the COPD‐related genes in the Chinese population. Therefore, we determined whether PRDX6 gene polymorphism was related to the genetic susceptibility of the Chinese Han population to COPD. We genotyped 4 tag SNPs and found one SNP site (rs33951697 site) that may be related to the risk of COPD, and this SNP site was reported for the first time in COPD. Our results indicated that the PRDX6 gene polymorphism may be pivotal in the pathogenesis of COPD. Sundar et al.^22^ proved through in vivo experiments that PRDX6 target targeting knockout did not aggravate lung inflammation, but it can increase the body's antioxidant enzyme levels, suggesting that PRDX6 played an antioxidant role in COPD.

Meanwhile, we analyzed the relationship between genotypes and clinical parameters in all selected SNPs, such as smoking behavior (cigarettes smoked per day and smoking packyears) and lung function. We found the number of cigarettes smoked per day and FEV1/FVC were statistically significant at PRDX6 rs4382766 and rs7314 with different genotypes.

Our findings expanded the current knowledge of PRDX6 and its role in the susceptibility to COPD. However, it was unclear whether rs33951697 polymorphism affected the normal cellular function of PRDX6. Moreover, the underlying mechanisms of the functional changes by mutated PRDX6 are not fully understood and remain to be further elucidated.

## 5 CONCLUSION

We found one SNP site, rs33951697, that may be related to the risk of COPD. Our study also suggested that PRDX6 polymorphisms and their interactions with smoking status may induce the occurrence of COPD. Moreover, smoking status seems to be no significant influence on the incidence of COPD. We also showed an association between polymorphisms in PRDX6 and lung function. However, the differences in mean age between the two groups might be a bias to influence the results, which should be avoided in future research. In addition, the sample scale may limit the accuracy of the experiment, and it is necessary to carry out large‐scale related research and functional research on PRDX6.

## AUTHOR CONTRIBUTIONS

Mingmei Xiong initiated the project and designed the study. Mingmei Xiong and Meihua Guo wrote the initial draft of the manuscript, performed most of the experiments, and conducted data analysis. Jing Li and Yan Zhou assisted in performing the experiments and in acquiring/analyzing data. Dongjian Huang participated in the discussion on experimental design and critically reviewed the manuscript.

## CONFLICT OF INTEREST STATEMENT

The authors declare no conflict of interest.

## ETHICS STATEMENT

This study was approved by The Ethics Committee of Third Affiliated Hospital of Guangzhou Medical University and all the other local Ethics committees.

## Data Availability

The data that support the findings of this study are available from the corresponding author upon reasonable request.
